# Efficacy of stinging nettle extract in combination with ε‐polylysine on the quality, safety, and shelf life of rainbow trout fillets

**DOI:** 10.1002/fsn3.2129

**Published:** 2021-01-13

**Authors:** Kazem Alirezalu, Milad Yaghoubi, Zabihollah Nemati, Boukaga Farmani, Amin Mousavi Khaneghah

**Affiliations:** ^1^ Department of Food Science and Technology Ahar Faculty of Agriculture and Natural Resources University of Tabriz Tabriz Iran; ^2^ Department of Food Science and Technology Faculty of Agriculture University of Tabriz Tabriz Iran; ^3^ Department of Animal Science Ahar Faculty of Agriculture and Natural Resources University of Tabriz Tabriz Iran; ^4^ Department of Food and Nutrition and Technology Faculty of Food Engineering University of Campinas Campinas Brazil

**Keywords:** natural antioxidant, rainbow trout, shelf life, stinging nettle (*Urtica dioica* L.), ε‐polylysine

## Abstract

The effects of incorporation of stinging nettle extract (3% and 6%) and ɛ‐polylysine (0.1% and 0.2%) on chemical, microbial properties, and stability of rainbow trout fish fillets wrapped in polyethylene bags (in atmosphere condition) and refrigerated for 12 days at 4°C were evaluated. No remarkable differences regarding the chemical composition of rainbow trout fish (protein, moisture, fat, and ash content) resulting from the treatments were noted. The lowest TBARS (thiobarbituric acid reactive substance) and the highest phenolic compounds were noted in samples treated with 6% SNE + 0.2% ɛ‐PL on day 12, while the highest inhibitory effects against the growth of TVC, psychrotrophic bacteria, coliform, yeast, and molds corresponded to samples treated with 6% SNE (T4 and T5) at day 12. During the storage, the samples' TVB‐N (total volatile base nitrogen) increased, whereas the total phenolic content of the rainbow trout samples declined. The rainbow trout samples treated with 6% SNE + 0.2% ɛ‐PL had the highest amount of redness and the lowest TVB‐N values. Therefore, these natural ingredients could be used to maintain rainbow trout meat quality and shelf life.

## Graphical abstract

Combined ɛ‐PL and SNE on quality properties of rainbow trout fillets were evaluated. ɛ‐PL in combination with SNE had better antioxidant and antimicrobial activities The sensory characteristics of samples was improved by combined ɛ‐PL and SNE. ɛ‐PL and SNE can be applied as a natural alternative to improve quality of rainbow trout.

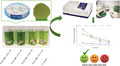

## 1 INTRODUCTION

Fish meat is a good source of essential amino acids, vitamins (D and E), minerals, and essential fatty acids such as ω6 and ω3, which can inhibit heart diseases (Kakani & Shahbazi, 2016). Oxidation reactions and microbial growth are among the most critical concerns in meat and meat products, decreasing quality indices, and shelf life (Alirezalu, Pateiro, et al., 2020). However, the synthetic antioxidants, due to high stability, low cost, and high efficiency, can be effectively used for further extension in the stability, shelf life, and improvements in quality properties of fish meat because of toxic sights and low overall acceptability among consumers, extensive studies have been investigated to find the alternative natural compounds (Agregán et al., 2019; Mahmoudzadeh et al., 2017; Tavakoli et al., 2019; Vargas et al., 2019). Natural antioxidative compounds and antimicrobials such as plant extracts (Alirezalu et al., 2017; Vincekovic et al., 2017), ε‐polylysine (Chang et al., 2014), and chitosan (Alirezalu et al., 2019) can effectively use in the meat industry to improve quality characteristics and shelf‐life stability.

ε‐polylysine (ɛ‐PL) produces as a result of a nonpathogenic microorganism's fermentation like *Streptomyces albulus* (Xu et al., 2018). It is a homopeptide consisting of 25 to 35 L‐lysine residues linked via isopeptide bonds between the α‐carboxyl and ɛ‐amino groups (Xu et al., 2018). Based on the previous research, ɛ‐PL absorbed electrostatically on the microorganism's cell surface, which eventually leads to the abnormal distribution in the cytoplasm and finally damages the microorganisms' cell wall (Shao et al., 2020). ɛ‐PL, as a nontoxic, biodegradable, water‐soluble and stable at high temperatures compound, poses antimicrobial effects against a broad spectrum of microorganisms such as yeasts and molds, viruses, *Staphylococcus aureus, Escherichia coli, Serratia marcescens*, and *Pseudomonas aeruginosa,* which is used in meat and meat products such as sea bass, beef, and chilled pork (Alirezalu et al., 2021; Cai et al., 2015; Chang et al., 2014).

Stinging nettle (*Urtica dioica* L.) as herbal medicine offers some properties including purgative, homeostatic, expectorant, vermifuge, and diuretic, anticancer, antirheumatism, antieczema, antihemorrhoids, antihyperthyroidism, and antibronchitis (Sharma et al., 2018). Martinez et al. (2006) showed that SNE has high inhibitory effects against microbial growth and can extend fresh pork sausage's functional characteristics. Plant leaves have not only high antioxidant and antimicrobial properties against a broad spectrum of bacteria's (Upton, 2013) but also is a good source of phenolic components, elements, tannins, flavonoids, and chlorophylls (Lorenzo & Munekata, 2016; Said et al., 2015). Aksu and Kaya (2004) reported that SNE significantly decreases the microbial count in Sucuk (Turkish dry‐fermented sausage). Furthermore, it is used as a natural and valuable antioxidant in the meat industry, such as beef patties (Akarpat et al., 2008), ground beef (Alp & Aksu, 2010), and frankfurter‐type sausage (Alirezalu et al., 2019). Some studies have individually focused on the antimicrobial and antioxidant properties of ɛ‐PL and SNE incorporated in different meat products. However, no previous studies regarding the synergistic effects of these natural preservatives on the quality characteristics and shelf life of trout meat during storage were conducted. Therefore, the current study was aimed to assess the effects of incorporation of ε‐PL combined with stinging nettle extract on the microbial and quality characteristics of rainbow trout fillet during 12 days of refrigerated storage.

## 2 MATERIALS AND METHODS

### 2.1 Chemical agents

All chemicals and microbial mediums with a purity of >99% (analytical grade) were purchased from Merck. ε‐polylysine powder with food‐grade purity was purchased from the FoodChem company (5,000 IU/ml).

### 2.2 Preparation of stinging nettle extracts and ε‐PL solutions

According to Ebrahimzadeh et al. (2008) method with some modification, nettle leaves (*Urtica dioica* L.) were collected in June 2018, from the Astara region (Gilan Province, Iran), dried in an oven at 40°C for 48 hr, and then sifted through 14‐inch sieves. 30 g from ground leaves was mixed by distilled water, reached 1,000 ml (3%), and 60 g was also reached 1,000 ml (6%). Finally, the obtained solution was shaken and set for 15 min in a water bath at 90°C and then filtered. ɛ‐PL stock solutions were prepared according to the method described by Hampikyan and Ugur (2007) with some modifications: Separately, 1 and 2 g of ɛ‐PL were solubilized in 2% acetic acid solutions with heating (60°C), reached to 1,000 ml, and sterilized by filtration through membrane filters (0.45 μm; Minisart, NML; Sartorius). The solutions were prepared immediately before use.

### 2.3 Preparation of meat

Fresh rainbow trout was purchased from a fish breeding pool in East Azarbaijan. The samples (ranged between 250 and 300 g) were transferred directly to the laboratory. The samples were slaughtered, washed without their skin removed, and then cut into 1 × 3 × 5 cm pieces. Minced samples (3 samples for each sampling point) were divided into five treatment (1 kg for each sample), immersed in SNE (1 hr at 4°C) and after drained the samples immersed in ɛ‐PL (35 min at 4°C) and packaged in polyethylene bags; T1: control; T2: 3% SNE + 0.1% ɛ‐PL; T3: 3% SNE + 0.2% ɛ‐PL; T4: 6% SNE + 0.1% ɛ‐PL; T5: 6% SNE + 0.2% ɛ‐PL. The samples were stored 12 days at the refrigerator (4°C) to evaluate quality properties, total phenolic content, pH, color indexes, TBARS, and microbial count at days 1, 4, 8, and 12 of storage.

### 2.4 Proximate composition and pH

Chemical composition (moisture, fat, ash, and protein) was measured as described by standard methods (AOAC, 1995). After calibration of the pH meter (Hanna; Methrom), trout samples (10 g) were homogenized by distilled water (100 ml) at a ratio of 1:10, and pH was measured.

### 2.5 Measurement of thiobarbituric acid reactive substances

Thiobarbituric acid reactive substances (TBARS) were determined by using a spectrophotometer. The blender for 30 s at 5000 g was used for homogenizing 10 g trout samples, 25 ml of trichloroacetic acid (20%), and 20 ml of distilled water. After centrifuging the mixture at  2000 g for 20 min, the Whatman No.1 filter was used for filtration. 2 ml of the filtrated solution was mixed with 2 ml of 0.02 M 2‐thiobarbituric acid in a glass tube. For 20 min, the tubes were heated at 100°C in a water bath and then cooled rapidly in running tap water for 5 min. The supernatant's absorbance was determined at 532 nm using a Hitachi U‐3210 spectrophotometer (Hitachi, Ltd.), and lipid oxidation were represented as mg malondialdehyde/kg fish (Faustman et al., 1992).

### 2.6 Determination of total volatile base nitrogen

Total volatile nitrogen (TVB‐N) of meat samples were evaluated by the Kjeldahl method with a vapor distillation, according to Goulas and Kontominas (2005). The data were reported as mg/100 g of chicken meat samples.

### 2.7 Determination of total phenolic content

Total phenolic content (TPC) of trout samples was determined spectrophotometrically, according to the Folin–Ciocalteau (F–C) reagent (Liu et al., 2009) is reported as follows. 50 g of trout meat was heated with 100 ml distilled water at 100°C for 20 min. After rapid cooling, samples were filtered in test tubes and mixed with 2.5 ml of Folin–Ciocalteau reagent and 5 ml of saturated sodium carbonate solution. Solutions were thoroughly mixed, held for one h in a dark room, and the supernatant's absorbance was recorded with UV‐VIS spectrophotometer JANEWAY 6405 (Bibby scientific Ltd.; Dunmow, Essex CM6 3LB) at 700 nm. Gallic acid was established as the standard curve and the results reported as mg gallic acid/100 g dry weight.

### 2.8 Determination of color values

According to Leon et al. (2006) method, a digital imaging method was established to determine sample internal and external color (L*: lightness, a*: red‐green index, and b*: yellow‐blue index). Samples were prepared in 10 × 30 × 30 mm thickness, and the camera with eight megapixels resolution was established by capturing the digital color image of the samples under calibrated lighting conditions at 20°C in three measurements. MATLAB (The Mathworks, Inc.; Version 6.1, United States) software was established to determined L*a*b* values of the fish samples.

### 2.9 Microbiological analysis

A portion of fish meat (25 g) was transferred to a blender bag and mixed with 0.1% sterile peptone water for 3 min and reached 200 ml. Pour plate method was utilized to enumerate bacteria with appropriate serial dilutions for each microorganism. Plate count agar (PC Agar), dichloran rose‐bengal chloramphenicol agar (DRBC Agar), violet red bile agar (VRB Agar), and King agar were used to report of total viable bacteria, mold, and yeast, coliform, and total psychrotrophic bacteria count, respectively. For enumerated total viable count, mold and yeast count, coliforms, and psychrotrophic bacteria counts, the medium was incubated at 30°C for 48–72 hr, 25°C for five days, 37°C for 48 hr, and 21°C for 48 hr, respectively. Microbial properties were reported as Log CFU/g of fish samples (FDA, 2013).

### 2.10 Statistical analysis

The statistical analysis of meat data was carried out according to statistical analysis software (SAS) (v.9, SAS Institute, United States America). Random block design, considering a mixed effect of sample treatments and refrigerated period (12 days) as fixed effects and three measurements as a random effect, was utilized for analyzing TVB‐N, TBARS, total phenolic and pH values, microbial count, and color data. The Tukey test compared one way ANOVA was also used to analyze the proximate composition and three observations. Statistical significance was indicated by *p* < .05 value, and all results were represented as mean values ± *SE*.

## 3 RESULTS AND DISCUSSION

### 3.1 Chemical composition and pH

The fat, moisture, and ash content ranged between 2.50% and 2.81%, 72.31 and 72.95%, and 1.84 and s2.50%, respectively. Therefore, the addition of SNE, combined with ε‐PL, had no significant effects (*p* > .05) on trout samples' proximate composition. Furthermore, there was no significant difference in protein content between control and treated meats (Table 1). Alirezalu et al. (2017) showed that stinging nettle, olive leaves, and green tea extracts added in frankfurter sausages had no significant effects on fat, ash, moisture, and protein content. Liu et al. (2009) and Hayes et al. (2010) evaluated natural plant extracts and reported similar results in chicken sausage and raw minced beef patties, respectively. Furthermore, Alirezalu et al. (2019) evaluated ε‐PL combined with plant extracts on the frankfurter‐type sausage stability. The authors showed that ε‐PL and plant extracts had no remarkable effects on chemical composition.

**TABLE 1:** Chemical composition of trout meat treated with combined SNE and ε‐PL at 4°C during storage

Trout samples	Properties (%)			
	Moisture	Fat	Ash	Protein
T1	72.95	2.81	1.84	21.80
T2	72.60	2.58	2.23	21.56
T3	72.36	2.50	2.50	22.22
T4	72.60	2.50	2.25	21.95
T5	72.31	2.58	2.19	22.39
*SEM*	0.142	0.188	0.198	0.232

Note

T1: Control; T2: 3% SNE + 0.1% ɛ‐PL; T3: 3% SNE + 0.2% ɛ‐PL; T4: 6% SNE + 0.1% ɛ‐PL; T5: 6% SNE + 0.2% ɛ‐PL. There were no significant differences among treatments.

The function of bacteriostatic and microbial equipoise in meat products is highly attributed to pH values. Therefore, pH value is an important chemical parameter of meat and meat products, influencing meat and meat products' safety and consumers' overall acceptability (Cullere et al., 2018). The pH of trout samples increased during the storage, which can be correlated with the microorganisms function (*Pseudomonas* and *Achromobacter* spp), consequently producing tri‐methyl amines in meat (Wang et al., 2017). Moreover, the accumulation of alkaline substances may be another reason for increasing pH during refrigerated storage (Radha Krishnan et al., 2014; Wang et al., 2017).

**FIGURE 1:**
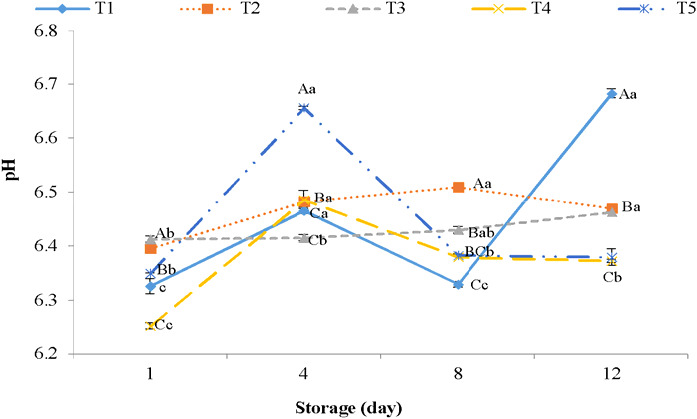
Changes in pH values of trout samples treated with combined SNE and ε‐PL at 4°C during storage. T1: Control; T2: 3% SNE + 0.1% ɛ‐PL; T3: 3% SNE + 0.2% ɛ‐PL; T4: 6% SNE + 0.1% ɛ‐PL; T5: 6% SNE + 0.2% ɛ‐PL. ^a‐c^ Mean during storage presented by a different letter is significantly different (*p* < .05). ^A‐C^ Mean between treatments presented by a different letter is significantly different (*p* < .05)

At the end of refrigerated storage, treated trout meats with 6% nettle extract (with 0.1% and 0.2% of ε‐PL) significantly (*p* < .05) had the lowest pH while compared with other groups especially control samples, due to high antimicrobial activities of nettle extract (Upton, 2013) and ε‐PL (Chang et al., 2014) (Figure 1). These results are paralleled with Mingyuan et al. (2020) reports, which showed that rosemary extract and ε‐PL incorporated in edible films significantly (*p* < .05) decreased the pH of ready‐to‐eat carbonado chicken during the storage period.

### 3.2 Measurement of thiobarbituric acid reactive substances and total volatile base nitrogen

Oxidation reactions, particularly in lipids, can remarkably affect stability and decline the shelf life of meat and meat products (Nemati et al., 2020). TBARS values as an efficient indicator in evaluating lipid oxidation in meat products associated with the accumulation of secondary products from lipid peroxides and hydro‐peroxides (Lorenzo et al., 2018).

Oxidation reactions in trout samples were significantly (*p* < .05) affected by the treatment of a combination of SNE and ε‐PL. TBARS value in all trout samples was remarkably increased within storage; however, the rate of this increase in treated samples was lower than control, which may be associated with antioxidant activity proposed by incorporating SNE and ε‐PL (Table 2). At day 1 and 4 of storage, there were no remarkable (*p* > .05) differences between treated and untreated trout samples; however, at the day of 8 and 12, there was a significant (*p* < .05) decrease in TBARS in treated samples with SNE and ε‐PL while compared with control (Table 2). On day 12 of refrigerated storage samples treated with 6% SNE (T4 and T5) had the lowest TBARS values significantly while compared with other treatments. Alp and Aksu (2010) demonstrated that 500 ppm SNE and MAP incorporated in ground beef could significantly decrease lipid oxidation during storage. Moreover, Alirezalu et al. (2019) evaluated SNE and chitosan effects on frankfurter‐type sausages' shelf life and indicated that these ingredients decrease lipid oxidation significantly during storage. Furthermore, Karabacak and Bozkurt (2008) also evaluated the antioxidant activity of SNE in Sucuk and reported similar results. The high antioxidant capacity of SNE may be caused by a high amount of phenolic components such as rutin, ferulic acid, naringin, kaempferol, myricetin, quercetin, ellagic acid, p‐coumaric acid, and isorhamnetin (Otles & Yalcin, 2012).

**TABLE 2:** Changes in TBARS and TVB‐N values of trout samples treated with combined SNE and ε‐PL at 4°C during storage

Parameters	Trout samples	Storage (day)			
		1	4	8	12
TBARS (mg/kg)	T1	0.56 ± 0.014^Ac^	0.70 ± 0.015^Ac^	1.17 ± 0.040^Ab^	1.55 ± 0.064^Aa^
	T2	0.52 ± 0.005^Ac^	0.59 ± 0.023^Abc^	0.72 ± 0.023^BCb^	1.23 ± 0.035^Ba^
	T3	0.55 ± 0.017^Ac^	0.57 ± 0.031^Ac^	0.83 ± 0.031^Bb^	1.15 ± 0.066^Ba^
	T4	0.50 ± 0.008^Ac^	0.54 ± 0.017^Abc^	0.71 ± 0.049^Cb^	0.93 ± 0.029^Ca^
	T5	0.50 ± 0.024^Ac^	0.54 ± 0.014^Abc^	0.68 ± 0.030^Cab^	0.780 ± 0.058^Ca^
TVB‐N (mg/100 g)	T1	8.88 ± 0.060^Ad^	22.76 ± 0.153^Ac^	39.36 ± 0.257^Ab^	55.76 ± 0.145^Aa^
	T2	8.59 ± 0.112^ABd^	22.36 ± 0.363^Ac^	37.27 ± 0.214^Bb^	55.83 ± 0.088^Aa^
	T3	7.38 ± 0.180^BCd^	17.63 ± 0.158^Bc^	28.20 ± 0.060^Db^	44.53 ± 0.145^Ba^
	T4	8.48 ± 0.127^ABd^	18.23 ± 0.033^Bc^	31.70 ± 0.251^Cb^	34.50 ± 0.173^Ca^
	T5	6.33 ± 0.088^Cd^	15.76 ± 0.088^Cc^	23.26 ± 0.560^Eb^	31.26 ± 0.735^Da^

Note

T1: Control; T2: 3% SNE + 0.1% ɛ‐PL; T3: 3% SNE + 0.2% ɛ‐PL; T4: 6% SNE + 0.1% ɛ‐PL; T5: 6% SNE + 0.2% ɛ‐PL.

^a‐d^Mean during storage presented by a different letter is significantly different (*p* < .05).

^A‐E^Mean between treatments presented by a different letter is significantly different (*p* < .05).

Total volatile base nitrogen value is an indicator for evaluating meat and meat products' freshness (Chaparro‐hernandez et al., 2015). Changes in TVB‐N values may be attributed to protein degradation by corrosion‐causing microorganisms and exudation of proteolytic enzymes, which lead to producing compounds with alkaline nitrogen (Li et al., 2019). The TVB‐N values increased remarkably in all samples during storage (Wang et al., 2017). On day 1, TVB‐N values of T5 and T1 were 6.33 and 8.88 mg/100 g sample, respectively, which after 12 days, reached 31.26, and 55.76 mg/100 g (Table 2). On day 12, the trout samples incorporated with 6% SNE + 0.2% ɛ‐PL significantly (*p* < .05) had the lowest amount of TVB‐N values. The high antimicrobial potency of ε‐PL and nettle leaves extract may be the main reason for low TVB‐N values. Lower TVB‐N values in trout samples incorporated with 6% SNE + 0.2% ɛ‐PL might show a lower microbial population, which agrees with Liu et al. (2009) results. Liu et al. (2020) showed that added ɛ‐PL, essential oils, and nisin in ready‐to‐eat Yao meat products could significantly delay increasing TVB‐N values during the storage period compared to control. Furthermore, Alirezalu et al. (2019) evaluated the effects of chitosan and ε‐PL incorporated with natural plant extracts and reported similar results.

### 3.3 Total phenolic content

**FIGURE 2:**
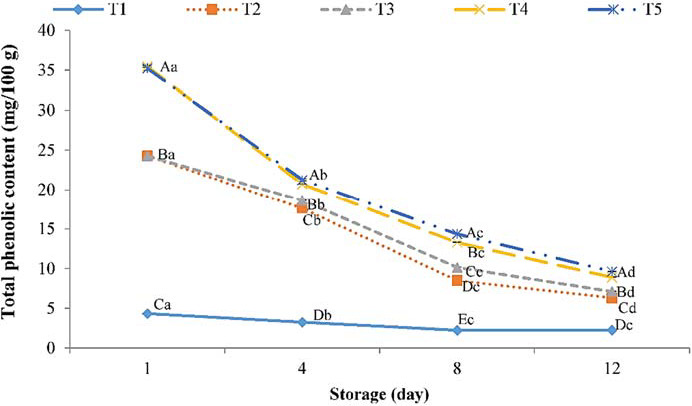
Changes in Total phenolic content (TPC) of trout samples treated with combined SNE and ε‐PL at 4°C during storage. T1: Control; T2: 3% SNE + 0.1% ɛ‐PL; T3: 3% SNE + 0.2% ɛ‐PL; T4: 6% SNE + 0.1% ɛ‐PL; T5: 6% SNE + 0.2% ɛ‐PL. ^a‐d^ Mean during storage presented by a different letter is significantly different (*p* < .05). ^A‐E^ Mean between treatments presented by a different letter is significantly different (*p* < .05)

Plants extracts contain high levels of phenolic compounds which possess high antimicrobial and antioxidant activities (Rusak et al., 2008), while also they can lead to healthier and higher levels of techno‐functional properties in meat and meat products (Alirezalu, Ahmadi, et al., 2020). During storage, the TPC of all trout meats was significantly (*p* < .05) declined. Oxidative reactions during storage may be the main reason for phenolic compound reduction (Daskalaki et al., 2009). On day 12, treated samples with 6% SNE + 0.2% ɛ‐PL reached 0.78 mg GA/ 100 g DW, whereas this number for control was 1.55 mg GA/ 100 g DW (Figure 2). High phenolic content in plant extract and its antioxidant properties may be the main reason for higher phenolic contents in treated samples at the end of storage compared with control (Rusak et al., 2008).

These findings are in agreement with, as described by Alirezalu et al. (2017). The authors evaluated the effects of natural plant extract (including stinging nettle, olive leaves, and green tea extracts) on the phenolic compounds of frankfurter‐type sausages and showed a similar reduction in phenolic compounds storage. Moreover, Liu et al. (2009) also evaluated the effects of natural plant extracts incorporated with fresh chicken sausage and reported similar results.

### 3.4 Color indexes

Color values in meat and meat products as one of the most critical sensory attributes parameters can significantly affect sensory properties, especially the overall acceptability of products (Zhang et al., 2007). Color values of trout meat were affected by SNE incorporated with ε‐PL (Table 3). Fat oxidation in meat and meat products has a strong relationship with redness (Fernandez‐Ginés et al., 2005). A decrease in meat redness may be caused by pro‐oxidative activity and heme pigments ability in iron, releasing positively affected color changes in meat (Kanner et al., 1991). During storage, a* value decreased in the control sample. However, in trout meats treated with SNE + ɛ‐PL, a* value increased probably due to the formation of the stable myoglobin‐phenol complex. On day 12, control displayed significantly (*p* < .05) the lowest amount of a^*^ value. The higher b^*^ value in treated samples with SNE and ε‐PL may be due to a dark color component in SNE, quinone formation, and meat pigments dilution (Ryu et al., 2014).

**TABLE 3:** Color indexes of trout meat treated with combined SNE and ε‐PL at 4°C during storage

Properties	Trout samples	Storage (day)			
		1	4	8	12
	T1	52.27 ± 0.19^Dab^	53.66 ± 0.38^ABa^	52.27 ± 0.19^ABab^	50.66 ± 0.48^Bb^
L*	T2	60.19 ± 0.06^Aa^	54.89 ± 0.06^Ab^	52.27 ± 0.36^ABc^	50.68 ± 0.26^Bd^
	T3	57.66 ± 0.91^ABa^	52.33 ± 0.58^Bb^	51.03 ± 0.58^Bbc^	50.19 ± 0.58^Bc^
	T4	55.66 ± 0.19^BCa^	54.77 ± 0.64^Aa^	54.16 ± 0.48^Aab^	53.22 ± 0.36^Ab^
	T5	54.27 ± 0.72^CDa^	53.43 ± 0.06^ABa^	53.11 ± 0.49^Aa^	53.94 ± 0.86^Aa^
	T1	−2.49 ± 0.09^Dc^	−3.28 ± 0.11^Bb^	−3.40 ± 0.20^Ab^	−4.49 ± 0.09^Aa^
a*	T2	−3.38 ± 0.14^Ca^	−3.37 ± 0.11^ABa^	−3.04 ± 0.11^Bb^	−2.92 ± 0.05^Bb^
	T3	−3.92 ± 0.24^Ba^	−3.57 ± 0.04^Ab^	−3.38 ± 0.04^Ac^	−3.1 ± 0.04^Bd^
	T4	−4.22 ± 0.22^Aa^	−3.55 ± 0.11^Ab^	−2.79 ± 0.10^Cc^	−2.29 ± 0.03^Cd^
	T5	−3.65 ± 0.003^Ca^	−3.61 ± 0.005^Aa^	−2.61 ± 0.10^Cc^	−1.13 ± 0.06^Cd^
	T1	28.54 ± 0.28^Aa^	28.54 ± 0.28^Aa^	26.42 ± 0.12^Aa^	23.72 ± 0.36^Bb^
b*	T2	22.98 ± 0.19^Cb^	22.72 ± 0.29^Cb^	27.50 ± 0.44^Aa^	28.37 ± 0.44^Aa^
	T3	25.33 ± 0.50^Bb^	26.20 ± 0.50^Bb^	27.19 ± 0.50^Aab^	28.94 ± 0.77^Aa^
	T4	25.40 ± 0.14^Bb^	25.55 ± 0.24^Bb^	28.33 ± 0.28^Aa^	28.38 ± 0.33^Aa^
	T5	27.61 ± 0.11^ABa^	27.33 ± 0.16^ABa^	28.10 ± 0.36^Aa^	28.59 ± 0.21^Aa^

Note

T1: Control; T2: 3% SNE + 0.1% ɛ‐PL; T3: 3% SNE + 0.2% ɛ‐PL; T4: 6% SNE + 0.1% ɛ‐PL; T5: 6% SNE + 0.2% ɛ‐PL.

^a‐d^Mean during storage presented by a different letter is significantly different (*p* < .05).

^A‐D^Mean between treatments presented by a different letter is significantly different (*p* < .05).

### 3.5 Microbiological tests

Total viable count (TVC) was remarkably (*p* < .05) in all trout samples and increased within storage at 4°C, particularly for control. The control sample showed the highest growth rate, which substantiates the possibility of using the ε‐PL in combination with SNE in prolonging the trout meats' shelf‐life stability. Table 4 shows that on day 12, treated samples with 3% SNE + 0.2% ɛ‐PL and control samples had the lowest (8.74 Log CFU/g) and highest (9.05 Log CFU/g) amount of TVC, respectively. Alirezalu et al. (2019) indicated that ε‐PL combined with SNE could significantly (*p* < .05) inhibit microbial growth in frankfurter‐type sausage. Furthermore, the authors showed chitosan (1%) + ɛ‐PL (0.2%) combined with plant extracts displayed a 30% longer shelf life in sausage samples. Coliforms as a hygienic quality indicator generally used in meat and meat products (Lorenzo et al., 2017). As shown in Table 4, coliforms increased remarkably (*p* < .05) during refrigerated storage. At the end of storage, samples incorporated with 6% SNE + 0.2% ɛ‐PL showed the lowest coliforms.

**TABLE 4:** Evaluation of microbiological count (Log CFU/g) in trout meat treated with combined SNE and ε‐PL at 4°C during storage

Microorganisms	Trout samples	Storage (day)			
		1	4	8	12
Total viable count	T1	3.59 ± 0.01^Ad^	5.39 ± 0.008^Ac^	8.00 ± 0.01^Ab^	9.05 ± 0.02^Aa^
	T2	3.18 ± 0.005^Bd^	5.36 ± 0.01^Ac^	7.88 ± 0.005^ABb^	8.87 ± 0.008^ABa^
	T3	3.12 ± 0.01^Bd^	5.27 ± 0.008^ABc^	7.61 ± 0.008^Bb^	8.74 ± 0.01^Ba^
	T4	3.24 ± 0.003^Bd^	5.05 ± 0.03^Bc^	7.84 ± 0.008^ABb^	8.90 ± 0.005^ABa^
	T5	3.12 ± 0.05^Bd^	4.60 ± 0.07^Cc^	7.59 ± 0.006^Bb^	8.77 ± 0.008^Ba^
Coliform	T1	0.86 ± 0.08^Ad^	1.96 ± 0.01^Ac^	2.35 ± 0.02^Ab^	3.25 ± 0.008^Aa^
	T2	0.69 ± 0.20^Bd^	1.73 ± 0.01^Ac^	2.84 ± 0.008^Bb^	3.07 ± 0.008^ABa^
	T3	0.23 ± 0.12^Cc^	1.64 ± 0.02^ABb^	2.83 ± 0.01^Ba^	2.94 ± 0.005^Ca^
	T4	0.25 ± 0.03^Cd^	1.32 ± 0.03^BCc^	2.80 ± 0.01^Bb^	3.98 ± 0.01^Ba^
	T5	0.14 ± 0.03^Cc^	1.00 ± 0.01^Cb^	2.81 ± 0.003^Ba^	2.87 ± 0.01^Ca^
Psychrotrophic bacteria	T1	2.63 ± 0.06^Ad^	3.42 ± 0.03^Ac^	5.43 ± 0.05^Ab^	7.74 ± 0.01^Aa^
	T2	2.48 ± 0.02^Bd^	3.33 ± 0.03^Ac^	5.39 ± 0.01^Ab^	7.38 ± 0.005^Aa^
	T3	2.22 ± 0.09^Cd^	3.26 ± 0.009^ABc^	4.64 ± 0.06^Bb^	6.11 ± 0.02^Ca^
	T4	2.26 ± 0.009^Cd^	3.39 ± 0.09^Ac^	5.52 ± 0.03^Ab^	6.82 ± 0.01^Ba^
	T5	2.21 ± 0.03^Cc^	3.01 ± 0.03^Bb^	4.33 ± 0.04^Ca^	4.36 ± 0.02^Da^
Mold and yeast	T1	2.29 ± 0.06^Ab^	5.17 ± 0.01^Aa^	5.19 ± 0.005^Aa^	5.27 ± 0.008^Aa^
	T2	1.84 ± 0.03^Bb^	5.02 ± 0.01^Aa^	5.18 ± 0.01^Aa^	5.21 ± 0.005^ABa^
	T3	1.59 ± 0.06^Bb^	5.00 ± 0.01^Aa^	4.93 ± 0.01^Aa^	4.94 ± 0.006^BCa^
	T4	1.10 ± 0.10^Cb^	4.89 ± 0.02^ABa^	4.92 ± 0.01^ABa^	5.00 ± 0.01^ABCa^
	T5	1.16 ± 0.16^Cb^	4.59 ± 0.03^Ba^	4.58 ± 0.02^Ba^	4.71 ± 0.02^Ca^

Note

T1: Control; T2: 3% SNE + 0.1% ɛ‐PL; T3: 3% SNE + 0.2% ɛ‐PL; T4: 6% SNE + 0.1% ɛ‐PL; T5: 6% SNE + 0.2% ɛ‐PL.

^a‐d^Mean during storage presented by a different letter is significantly different (*p* < .05).

^A‐D^Mean between treatments presented by a different letter is significantly different (*p* < .05).

The meat surface is a highly susceptible place for microbial growth and increasing yeasts and molds as psychrotrophic bacteria, leading to decreased sensory scores. Psychrotrophic bacteria counts in all trout samples increased during 12 days of storage, and T1, T2, and T4 samples were reached the maximum acceptable limit before day 8, while the count of these bacteria in the treated sample with 6% SNE + 0.2% ɛ‐PL (T5) was below the proposed acceptable limit at the end of the storage period being significantly differences (*p* < .05) lower than other trout meats. On day 1, trout samples incorporated with ε‐PL and SNE displayed remarkably lower yeast and molds than control. Yeast and molds were increased significantly (*p* < .05) within storage at 4°C in all trout samples. Samples treated with 6% SNE + 0.2% ɛ‐PL had the lowest yeast count and molds at the end of storage (Table 4).

## 4 CONCLUSION

The findings of the present study demonstrated that SNE, in combination with ɛ‐PL, has significant antimicrobial properties and can increase the oxidative stability and extend significantly (*p* < .05) shelf life of the trout meats. The samples with 6% SNE (T4 and T5) had the lowest TBARS on day 12 (the end of storage) when compared to SNE 3% (T2 and T3). Moreover, samples with 6% SNE + 0.2% ɛ‐PL had the highest amount of total phenolic content with the lowest TVB‐N level compared to other treatments. Furthermore, 3% and 6% SNE combined with 0.2% ɛ‐PL displayed significantly (*p* < .05) highest inhibitory effects against TVC, yeast and molds, coliform, and psychrotrophic bacteria. Based on the obtained results, 6% SNE combined with 0.2% ɛ‐PL effectively extended the trout meat's safety and shelf life. Therefore, these ingredients could be used usefully for maintaining trout meat's quality and shelf life.

## CONFLICT OF INTEREST

The authors reported no potential conflict of interest.

## ETHICAL APPROVAL

This article does not cover any human or animal studies conducted by any of the authors.

## Data Availability

The data that support the findings of present study are available from the corresponding authors by reasonable request.
